# Efficacy of sodium bicarbonate ingestion strategies for protecting blinding

**DOI:** 10.1007/s00421-022-05031-0

**Published:** 2022-09-02

**Authors:** William H. Gurton, Guilherme G. Matta, Lewis A. Gough, Philip Hurst

**Affiliations:** 1 School of Psychology and Life Sciences, Canterbury Christ Church University, Canterbury, UK; 2 Sport and Physical Activity Research Centre, College of Health, Wellbeing and Life Sciences, Sheffield Hallam University, Sheffield, UK; 3 Human Performance and Health Research Group, Centre for Life and Sport Sciences, Birmingham City University, Birmingham, UK

**Keywords:** Dietary supplements, Double-blind, Sports nutrition, Research methods

## Abstract

Sodium bicarbonate (NaHCO_3_) is a widely researched ergogenic aid, but the optimal blinding strategy during randomised placebo-controlled trials is unknown. In this multi-study project, we aimed to determine the most efficacious ingestion strategy for blinding NaHCO_3_ research. During study one, 16 physically active adults tasted 0.3 g kg^−1^ body mass NaHCO_3_ or 0.03 g kg^−1^ body mass sodium chloride placebo treatments given in different flavour (orange, blackcurrant) and temperature (chilled, room temperature) solutions. They were required to guess which treatment they had received. During study two, 12 recreational athletes performed time-to-exhaustion (TTE) cycling trials (familiarisation, four experimental). Using a randomised, double-blind design, participants consumed 0.3 g kg^−1^ body mass NaHCO_3_ or a placebo in 5 mL kg^−1^ body mass chilled orange squash/water solutions or capsules and indicated what they believed they had received immediately after consumption, pre-TTE and post-TTE. In study one, NaHCO_3_ prepared in chilled orange squash resulted in the most unsure ratings (44%). In study two, giving NaHCO_3_ in capsules resulted in more unsure ratings than in solution after consumption (92 vs 33%), pre-TTE (67 vs. 17%) and post-TTE (50 vs. 17%). Administering NaHCO_3_ in capsules was the most efficacious blinding strategy which provides important implications for researchers conducting randomised placebo-controlled trials.

## Introduction

For more than 70 years, the double-blind, randomised placebo-controlled trial (RCT) has been regarded as the gold standard approach for testing treatment efficacy (Bothwell and Podolsky 2016). An important characteristic of RCTs is concealing participants’ allocation to the treatment under investigation and an equivalent placebo (Schulz and Grimes 2002). Blinding participants (single-blind) or participants and researchers (double-blind) to what has been administered can help ensure outcome variables are not influenced by biases, such as placebo effects, experimenter effects or self-report biases (Penić et al. 2020; Beedie et al. 2018; Hróbjartsson et al. 2007). If a participant is aware that they received the ‘real’ treatment, they may report more favourable outcomes and/or be more likely to adhere to using that treatment (and vice versa for a placebo) (Beedie et al. 2007; Hurst et al. 2020). For researchers conducting RCTs examining ergogenic aids, it is therefore essential participants are unable to identify treatments being administered.

One treatment that has received extensive attention in sport and exercise science is sodium bicarbonate (NaHCO_3_) (Peeling et al. 2018; Maughan et al. 2018). Ingestion of 0.2–0.3 g kg^−1^ body mass (BM) NaHCO_3_ enhances extracellular buffering capacity that in turn reduces acidity within active musculature during intense exercise (Bishop et al. 2004), which may improve high-intensity, short duration exercise performance (Hadzic et al. 2019). Ergogenic benefits can also be attributed to the belief that NaHCO_3_ improves performance (Higgins and Shabir 2016; McClung and Collins 2007), therefore adopting the most efficacious ingestion strategy for blinding NaHCO_3_ is crucial when evaluating treatment effectiveness. To our knowledge, however, no research has investigated the optimal strategy for blinding NaHCO_3_ during RCTs examining sports performance.

Blinding NaHCO_3_ during RCTs can prove difficult due to the ‘strong aftertaste/salty flavour’ and gastrointestinal discomfort after ingestion (Carr et al. 2011). To overcome these issues, researchers dissolve NaHCO_3_ in solutions of mineral water/squash and use sodium chloride as a placebo to replicate the ‘salty’ taste or to match sodium content between treatments (Deb et al. 2018). Alternatively, they administer NaHCO_3_ in capsules to reduce severity of gastrointestinal discomfort (Carr et al. 2011) and give cornflour in placebo capsules to match treatment appearance (de Oliveira et al. 2020). These approaches are used to blind NaHCO_3_ supplementation during RCTs examining sports performance (Gough et al. 2021; Gurton et al. 2021a), but few researchers have comprehensively assessed blinding efficacy to determine whether participants are able to identify which treatment they received. Gurton et al. (2020) reported that dissolving 0.3 g kg^−1^ BM NaHCO_3_ in mineral water/orange squash protected blinding during cycling time-to-exhaustion (TTE) trials. However, as blinding questionnaires were completed immediately post-consumption, it is unclear whether gastrointestinal side-effects after NaHCO_3_ influenced participants’ treatment assignment prior to TTE cycling.

To progress understanding of the ergogenic benefits associated with NaHCO_3_, it is important to identify the most efficacious ingestion strategy for blinding NaHCO_3_ during RCTs sports performance. Therefore, in a multi-study project we aimed to i) determine an optimal solution ingestion strategy that can be used during RCTs to taste-match NaHCO_3_ and a sodium chloride placebo (study 1), and ii) compare blinding efficacy of solution and capsule NaHCO_3_ ingestion strategies immediately after consumption to post-TTE cycling (study 2).

## Methods

### Experimental design

Block randomised, repeated measures, double-blind, placebo-controlled, counterbalanced, crossover experimental designs were employed for both studies. Participants from study 1 visited the laboratory on four occasions to taste eight treatments (four NaHCO_3_, four placebo). Participants from study 2 attended five laboratory visits to perform TTE cycling trials (familiarisation, four experimental). To control for order effects, treatments were randomly assigned to each visit in a balanced fashion during studies 1 and 2 using 8 × 2 and 4 × 4 Latin square sequences, respectively, by a member of the research team not involved with data collection.

## Study 1

### Participants

Sixteen physically active adults (10 male, 6 female; body mass, 76.6 ± 12.5 kg; age, 38.5 ± 10.4 years) volunteered for study 1. Inclusion criteria stipulated that participants needed to be aged 18–55 years and had never used NaHCO_3_ during training or competition. Ethical approval was gained from the Institutional Ethics Committee (ETH2021-0198). Research procedures were conducted in accordance with the revised Declaration of Helsinki (2014). Participants provided informed consent prior to commencing study procedures.

### Supplementation protocol

Participants received 0.3 g·kg^−1^ BM NaHCO_3_ (SB; Health Leads Ltd, UK) and 0.03 g·kg^−1^ BM sodium chloride placebo (PL; Sainsbury’s, UK) on four occasions (i.e., eight different treatments distributed across four sessions; two per visit). Treatments were weighed out using a biochemistry balance (± 0.001; AE Weighing Scales, USA) and dissolved in 4 mL·kg^−1^ BM water, before 1 mL·kg^−1^ BM of double strength orange or blackcurrant squash (Sainsbury’s, UK) was added. A fluid volume of 5 mL·kg^−1^ BM was chosen to replicate Gurton et al. (2020) where NaHCO_3_ and sodium chloride were successfully blinded. Orange and blackcurrant squash are common flavours used for taste-matching NaHCO_3_ and a placebo during RCTs. Treatments were chilled for 1 h (~ 12 °C) or left at room temperature (~ 18 °C) with colder solutions thought to improve palatability (Burdon et al. 2012). Pilot testing revealed that 0.03 g·kg^−1^ BM sodium chloride provided the best taste-match with 0.3 g·kg^−1^ BM NaHCO_3_, whereas other doses (0.07 g·kg^−1^ and 0.21 g·kg^−1^ BM) were too ‘salty’. Treatments were prepared by a member of the research team not involved with data collection and administered in opaque bottles to prevent participants from visually distinguishing between them.

### Experimental procedures & questionnaires

During each of the four sessions, participants tasted two different treatments, with water given in between to remove any aftertaste. They were required to take 2–3 sips to prevent gastrointestinal side-effects from influencing treatment assignment (i.e., aim was to identify closest taste-match for use during study 2). Treatment assignment questionnaires were completed after tasting each treatment that asked participants to select which treatment they thought had been given (“NaHCO_3_”, “placebo”, “unsure”) and explain reasons for their decision (Gurton et al. 2021b). Treatment palatability was assessed using 9-point Likert type scales validated for measuring food preferences (Peryam and Pilgrim 1957) anchored by “1” (extremely disliked) to “9” (extremely liked).

## Study 2

### Participants

Sixteen recreationally trained athletes were screened for eligibility; two did not meet inclusion criteria, one withdrew because of injury and one withdrew due to gastrointestinal discomfort after NaHCO_3_ ingestion. Therefore, a total of 12 participants (9 males, 3 females; stature, 176.3 ± 5.6 cm; body mass, 69.4 ± 8.1 kg; age, 29.3 ± 6.7 years; maximal oxygen consumption, 54.4 ± 5.7 mL·kg^−1^·min^−1^) volunteered for study 2. Inclusion criteria were recreationally trained adults, aged 18–40 years, performing > 3 h of running/cycling training per week. Participants were excluded if they had any preconceptions of NaHCO_3_ (e.g., awareness of gastrointestinal side-effects), used NaHCO_3_ in last 6 months, an intolerance to cornflour/lactose and a medical condition that could impact high-intensity exercise. Female participants recorded menstrual cycle (using a calendar-based method) to ensure experimental trials occurred during the same phase (follicular: 1–14 d, or luteal: 14 d to start of next cycle). Ethical approval was gained from the institutional ethics committee as per study 1 (ETH2021-0198). All participants provided written informed consent.

### Graded exercise test & familiarisation

On arrival to the laboratory during visit 1, baseline anthropometric measures were recorded, before participants performed a graded exercise test on an electronically braked SRM cycle ergometer (Schoberer Rad Meßtechnik, Germany). Gaseous exchange was collected using a breath-by-breath metabolic analyser (Vyntus CPX, CareFusion GmbH, Germany). Participants completed a 5 min warmup at 70 W and a self-selected cadence. Power output during the first stage was set according to fitness level, with increments of + 5 W every 15 s until volitional exhaustion. Average power output from the final 2 min was used as workload for TTE cycling (Saunders et al. 2013). Maximal oxygen consumption was defined as the highest 30 s average for oxygen uptake.

Following 30 min recovery, participants were familiarised to TTE cycling. Participants selected bike dimensions (which were repeated during subsequent visits) and completed a 5 min warmup at 1.5 W·kg^−1^ BM. Power output was then increased across 60 s (increments every 15 s) until desired workload was achieved, at which point participants commenced TTE cycling. Total elapsed time was blinded from participants and exercise was terminated if participants failed to maintain cadence > 60 rev·min^−1^ for 5 s despite verbal encouragement.

### Supplementation protocol

During visits 2–5, participants ingested one of four treatments 90 min prior to exercise: i) 0.3 g·kg^−1^ BM NaHCO_3_ in 5 mL·kg^−1^ BM solution (SB-SOL), ii) 0.03 g·kg^−1^ BM sodium chloride in 5 mL·kg^−1^ BM solution (placebo; PL-SOL), iii) 0.3 g·kg^−1^ BM NaHCO_3_ within size 0 vegetarian capsules (SB-CAP), or iv) an equal number of identical capsules that contained cornflour (placebo; PL-CAP). Treatments given in solution were prepared in 4 mL·kg^−1^ BM orange squash and 1 mL·kg^−1^ BM water, before being chilled (~ 12 °C) for 1 h prior to consumption. This solution ingestion strategy provided the best blind during study 1. All capsules (Your Supplements, Stockport, UK) were manually filled using a capsule filling device (ALL-IN Capsule, USA). Each capsule contained approximately 0.9 g NaHCO_3_ (Health Leads Ltd, UK) or 0.4 g cornflour (Sainsbury’s, UK). Capsules were checked for weight and administered (to the nearest whole capsule, 24 ± 3) in an equal volume of chilled orange squash/water to solution trials. Treatments were prepared by a member of the research team not involved with data collection and solution treatments were administered in opaque bottles as per study 1. Participants co-ingested treatments alongside a carbohydrate-rich meal (1.5 g·kg^−1^ BM carbohydrates; toasted bread/jam, cereal bars) to minimise the risk of gastrointestinal discomfort after NaHCO_3_ (Carr et al. 2011).

### Experimental procedures & questionnaires

Participants attended the laboratory in a 3 h post-prandial state having avoided strenuous exercise and replicated nutritional intake (via self-report diaries) for 24 h. Experimental trials were separated by 3–7 d to allow for appropriate recovery/washout and testing was conducted at a similar time of day (± 2 h) to control for confounding effects of circadian rhythms on exercise performance (Reilly 1990). Upon arrival to the laboratory, participants consumed treatments across 10 min, before completing treatment assignment questionnaires and scoring palatability. Using a 9-point Likert scale, participants also scored how likely they would be to use each treatment if they knew it would improve performance (“1” extremely unlikely to “9” extremely likely). Treatment assignment questionnaires were repeated pre-TTE cycling. Exercise commenced 90 min post-consumption of treatments and TTE cycling tests were completed as per familiarisation. Participants received no feedback on TTE cycling performance. Immediately post-TTE cycling, final treatment assignment questionnaires were completed. At no point were participants able to refer to previous treatment assignment answers when completing questionnaires.

## Statistical analysis

Percentages for correct, incorrect, and unsure ratings were calculated for all treatments during both studies. Ratings of treatment assignment were analysed using 2 × 2 Chi-square tests (*χ*^2^) to determine blinding efficacy (primary outcome). Cramer V statistic (*V*) is reported as the effect size for treatment assignment and interpreted as: > 0.05 (weak), > 0.10 (moderate), > 0.15 (strong) and > 0.25 (very strong) (Akoglu 2018). Shapiro–Wilk tests indicated that normal distribution was violated for ‘treatment palatability’ and ‘how likely participants would be to use treatment’ scores (secondary outcomes). Friedman tests were therefore conducted to examine differences between treatments, with median and *Z* values calculated. Significance values for pairwise comparisons were adjusted with Bonferroni correction factors to minimise type I error. Non-normally distributed effect sizes (*r*) for differences between treatments were calculated from Z/√n, with 0.10, 0.24 and 0.37 representing small, medium, and large effects, respectively (Ivarsson et al. 2013). All data were analysed using SPSS version 26 (IBM, Chicago, IL, USA) and the α-level of statistical significance was set at *p* < 0.05.

## Results

### Study 1: blinding and palatability of solution ingestion strategies

No differences in treatment assignment were observed between SB and PL prepared in room temperature orange squash (*χ*^*2*^(1) = 1.13; *p* = 0.288; *V* = 0.19), chilled orange squash (*χ*^*2*^(1) = 0.13; *p* = 0.723; *V* = 0.06), or room temperature blackcurrant squash (*χ*^*2*^(1) = 0.13; *p* = 0.723; *V* = 0.06). Differences in treatment assignment were reported for chilled blackcurrant squash, with more correct ratings for SB vs. PL (*χ*^*2*^(1) = 4.50; *p* = 0.034; *V* = 0.38). Preparing treatments with chilled orange squash resulted in highest % of unsure ratings and the fewest correct ratings, with 44% and 50% of participants identifying SB and PL, respectively. All ratings for treatments are presented in Table 1.

**Table 1:** Treatment assignment ratings for each solution ingestion strategy from study 1

	SB + ORT	SB + OC	SB + BRT	SB + BC	PL + ORT	PL + OC	PL + BRT	PL + BC
Correct ratings	10 (63%)	**7 (44%)**	8 (50%)	11 (69%)	**7 (44%)**	8 (50%)	9 (56%)	**5 (31%)**
Incorrect ratings	3 (19%)	4 (25%)	6 (38%)	4 (25%)	4 (25%)	4 (25%)	4 (25%)	4 (25%)
Unsure ratings	3 (19%)	5 (31%)	2 (13%)	1 (6%)	5 (31%)	4 (25%)	3 (19%)	7 (44%)

*ORT*  orange squash/water at room temperature; *OC* chilled orange squash/water; *BRT* = blackcurrant squash/water at room temperature; *BC* chilled blackcurrant squash/water. Percentage of treatment assignment ratings displayed in parenthesis. Number of correct guesses that were less than expected by chance alone (< 50%) are highlighted in bold

Solution ingestion strategy impacted treatment palatability for SB treatments (*χ*^*2*^(3) = 10.406; *p* = 0.015). Palatability was lower when SB was administered in chilled blackcurrant squash vs. chilled orange squash (Median: 2.5 vs. 4.0; *Z* = – 1.250; *p* = 0.037; *r* = 0.31). There were no differences in palatability score between PL treatments (all *p* > 0.05).

### Study 2: blinding and palatability of sodium bicarbonate

No differences in treatment assignment were reported between NaHCO_3_ and equivalent placebo at any time point (*p* > 0.05). Greater treatment assignment was observed for SB-SOL vs. SB-CAP, with the number of correct ratings higher for SB-SOL post-consumption (*χ*^*2*^(1) = 6.316; *p* = 0.012; *V* = 0.51), pre-TTE cycling (*χ*^*2*^(1) = 4.196; *p* = 0.041; *V* = 0.418) and post-TTE cycling (*χ*^*2*^(1) = 4.196; *p* = 0.041; *V* = 0.418). Administering treatments in capsules resulted in a greater % of unsure ratings and fewer correct ratings than solution, with the number of correct ratings for SB-CAP and PL-CAP less than the 50% expected by chance alone. All ratings for treatments are presented in Table 2. Most common reasons referenced by participants correctly identifying treatments at each time-point are shown in Table 3.

**Table 2:** Treatment assignment ratings for NaHCO_3_ and placebo during study 2

	After consumption				Pre-TTE cycling				Post-TTE cycling			
	SB-SOL	SB-CAP	PL-SOL	PL-CAP	SB-SOL	SB-CAP	PL-SOL	PL-CAP	SB-SOL	SB-CAP	PL-SOL	PL-CAP
Correct ratings	**5 (42%)**	**0 (0%)**	6 (50%)	3 (25%)	8 (67%)	**3 (25%)**	7 (58%)	**3 (25%)**	9 (75%)	**4 (33%)**	7 (58%)	**4 (33%)**
Incorrect ratings	3 (25%)	1 (8%)	2 (17%)	2(17%)	2 (17%)	1 (8%)	1 (8%)	2 (17%)	1 (8%)	2 (17%)	3 (25%)	1 (8%)
Unsure ratings	4 (33%)	11 (92%)	4 (33%)	7 (58%)	2 (17%)	8 (67%)	4 (33%)	7 (58%)	2 (17%)	6 (50%)	2 (17%)	7 (58%)

SB-SOL = NaHCO_3_ in solution; SB-CAP = NaHCO_3_ in capsules; PL-SOL = placebo in solution; PL-CAP = placebo in capsules. Percentage of treatment assignment ratings displayed in parenthesis. Number of correct guesses that were less than expected by chance alone (< 50%) are highlighted in bold

**Table 3:** Most common factors referenced by participants correctly identifying treatments during study 2

	After consumption	Pre-TTE cycling	Post-TTE cycling
SB-SOL	Bad taste/fizzy	Side-effects	Better performance
SB-CAP	No correct guesses	Side-effects	Better performance
PL-SOL	Sweet/good taste	Absence of side-effects	Absence of side-effects
PL-CAP	No taste	Absence of side-effects	Worse performance

SB-SOL = NaHCO_3_ in solution; SB-CAP = NaHCO_3_ in capsules; PL-SOL = placebo in solution; PL-CAP = placebo in capsules

**Fig. 1:**
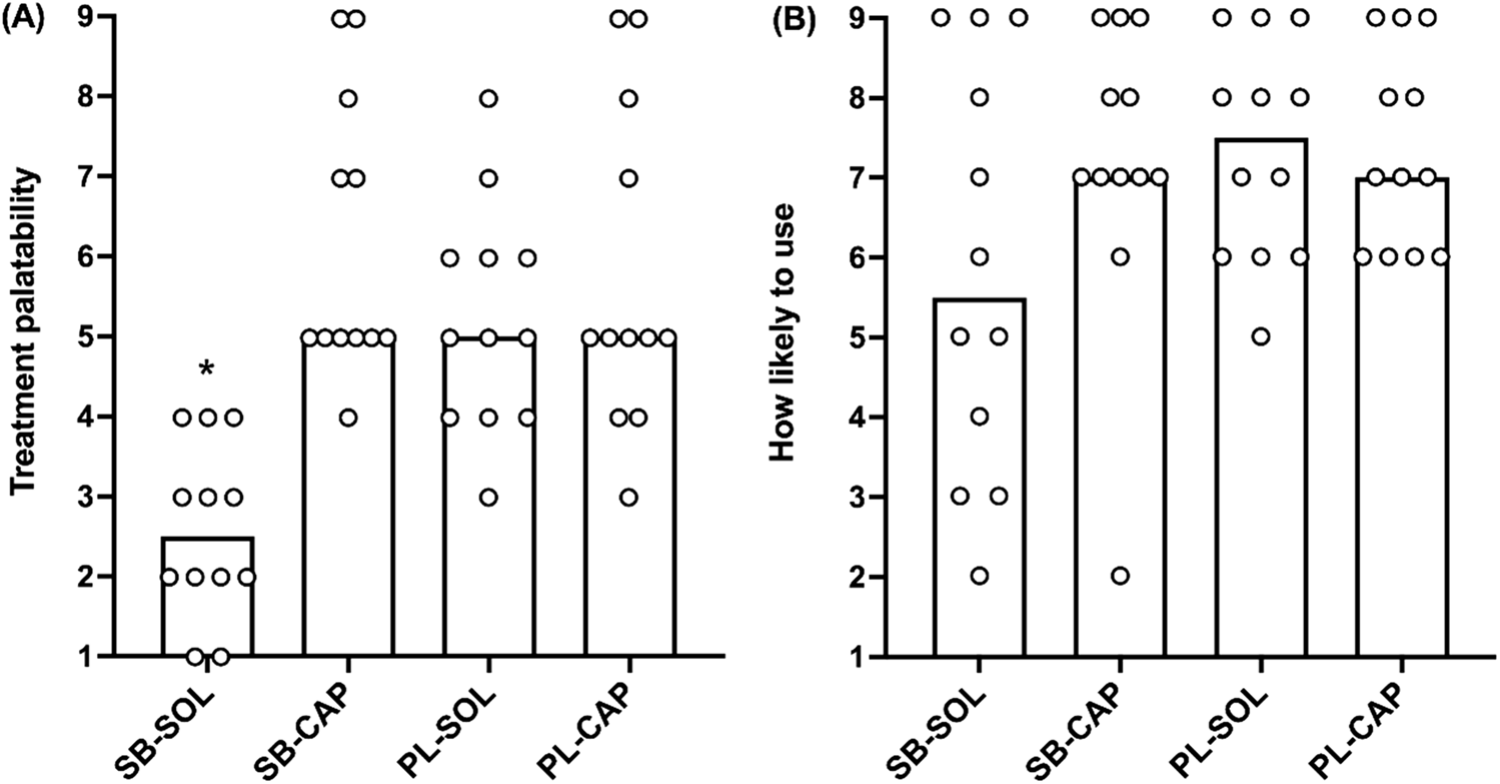
**A–B** Median and individual responses for palatability (**A**) and how likely to use (**B**) scores for NaHCO_3_ and placebo from study 2. *Note:* SB-SOL = NaHCO_3_ in solution; SB-CAP = NaHCO_3_ in capsules; PL-SOL = placebo in solution; PL-CAP = placebo in capsules. *SB-SOL vs. SB-CAP, PL-SOL & PL-CAP (*p* < 0.05)

Palatability scores revealed that all participants (100%) ‘disliked’ SB-SOL, whilst one (8%) ‘disliked’ SB-CAP. Four participants (33%) suggested that they would be ‘unlikely’ to use SB-SOL during training and/or competition, whereas one (8%) was ‘unlikely’ to use SB-CAP. Ingestion strategy significantly impacted palatability (*χ*^*2*^(3) = 22.294; *p* < 0.001), but not how likely participants would be to use treatments (*χ*^*2*^(3) = 6.83; *p* = 0.078). Palatability score was lowest for SB-SOL compared with SB-CAP (*Z* = –2.167; *p* < 0.001; *r* = 0.63), PL-SOL (*Z* = –1.417; *p* = 0.043; *r* = 0.41) and PL-CAP (*Z* = –1.917; *p* = 0.002; *r* = 0.55). There were no differences in palatability score for PL-CAP vs. SB-CAP (*Z* = 0.250; *p* = 1.000; *r* = 0.07), PL-SOL vs. SB-CAP (*Z* = 0.750; *p* = 0.928; *r* = 0.22) and PL-SOL vs. PL-CAP (*Z* = –0.500; *p* = 1.000; *r* = 0.14). Median and individual responses for palatability and how likely participants would be to use treatments are shown in Fig. 1 A–B.

## Discussion

We aimed to determine an optimal solution ingestion strategy that can be used during RCTs to taste-match NaHCO_3_ and a sodium chloride placebo (study 1) and compare blinding efficacy of solution and capsule NaHCO_3_ ingestion strategies immediately after consumption to post-TTE cycling (study 2). Chilled orange squash/water was the most efficacious solution ingestion strategy for blinding treatments during study 1, with the highest % of unsure ratings. Administering NaHCO_3_ in capsules improved blinding efficacy compared to solution ingestion during study 2, with greater treatment assignment and more correct ratings when NaHCO_3_ was given in solution compared to capsules. Differences in taste/fizziness and gastrointestinal discomfort were referenced by participants when identifying treatments. These results have important implications for researchers conducting placebo-controlled trials examining sports performance and suggest the need to adopt capsule NaHCO_3_ ingestion strategies to ensure an unbiased assessment of treatment efficacy.

### Optimising solution ingestion strategy

Chilled orange squash was the most efficacious solution ingestion strategy for taste-matching treatments during study 1, with the highest % of unsure ratings (Table 1). Participants acknowledged the ‘salty’ taste but were unable to consistently distinguish between treatments (i.e., 25% of participants incorrectly rated NaHCO_3_ as the sodium chloride placebo). Participants who identified treatments from study 1 suggested that NaHCO_3_ had a ‘bad aftertaste’, whilst the sodium chloride placebo had a ‘squash taste’. NaHCO_3_ prepared in chilled orange squash was the most pleasant tasting treatment, whereby palatability was higher than with chilled blackcurrant squash. Since cold (0–10 °C) or cool (10–22 °C) drinks have greater palatability (Burdon et al. 2012), it was unexpected that consuming NaHCO_3_ in chilled blackcurrant squash was the worst tasting treatment. Some participants suggested chilled orange squash treatments tasted like commercial sports drinks, therefore differences in palatability might be due to personal preferences. Administering NaHCO_3_ in chilled blackcurrant squash was the weakest blinding strategy, with 69% of participants identifying NaHCO_3_. In contrast, only 31% of participants correctly identified the equivalent sodium chloride placebo, which supports findings that breaking of blinding for a ‘real’ treatment exceeds a placebo (Hughes and Krahn 1985). Discrepancies in these accuracy ratings may represent a response bias (Margraf et al. 1991), as participants feel they are more likely to be given active treatments than a placebo.

Whilst study 1 aimed to evaluate the efficacy of solution ingestion approaches commonly used by researchers to blind NaHCO_3_ during RCTs examining sports performance, we did not account for the effect of carbonation on blinding. Upon being dissolved in water, NaHCO_3_ dissociates into bicarbonate ions, which in the presence of acids creates carbon dioxide gas. Both the orange and blackcurrant squash used during the present research contained citric acid, resulting in NaHCO_3_ treatments being fizzer. Best efforts were made to reduce the influence of fizziness on treatment assignment (i.e., opening the bottle away from participants so they could not hear the ‘fizz’), but a few participants correctly linked the carbonated sensation to NaHCO_3_. Despite this, in study one, only 3/16 participants correctly identified two NaHCO_3_ treatments due to fizziness and no participants identified all four treatments. Carbonated beverages are thought to have an acidic taste, as carbon dioxide gas is detected by sour-sensing cells on the tongue (Mielby et al. 2018). The fizzy sensations associated with NaHCO_3_ treatments were likely caused by diluted carbonic acid inducing a slight burning sensation that was detected via a mechanism known as chemesthesis (Simons et al. 1999). Carbonated water is thought to also stimulate lingual nociceptors via a carbonic anhydrase-dependent process, which in turn excites neurons responsible for these chemesthetic sensations (Simons et al. 1999). Therefore, preparing the sodium chloride placebo with carbonated water instead of mineral water might have replicated the fizzy sensation of NaHCO_3_ and improved blinding efficacy of solution treatments within the small number of participants who reported fizziness.

### Comparison between solution and capsule ingestion strategies

Efficacy of blinding was further strengthened by administering treatments in capsules during study 2, with a greater % of unsure ratings for NaHCO_3_ and cornflour placebo given in capsules compared to equivalent solution treatments at each time-point (Table 2). Very strong effect sizes were reported in favour of greater treatment assignment for NaHCO_3_ administered in solutions compared to capsules (*V* = 0.42–0.51). Previous findings from Gurton et al. (2020) suggest that preparing treatments in 5 mL·kg^−1^ BM of chilled orange squash/water protected blinding after consumption, but during the present study 42% and 50% of participants identified NaHCO_3_ and placebo solution treatments, respectively. These discrepancies could be attributed to solution flavour due to differences in sodium chloride dose (i.e., 0.3 g·kg^−1^ vs. 0.07 g·kg^−1^ BM). In contrast to solution treatments, no participants were able to identify NaHCO_3_ capsules immediately after consumption, likely as there was no taste/fizzy sensation to influence treatment assignment (Table 3), confirming that capsule NaHCO_3_ ingestion strategies improves blinding efficacy.

Treatment assignment changed from after consumption to pre-TTE cycling, such that 67% and 58% of participants identified NaHCO_3_ and placebo treatments given in solution, respectively. The percentage identifying NaHCO_3_ administered in solution was greater than 50% expected by chance alone, therefore any ergogenic benefits could partly be attributed to participant bias (Beedie et al. 2007). At the same time-point, only 25% of participants identified NaHCO_3_ capsules, which is less than previous research where 40% of participants identified NaHCO_3_ administered in capsules prior to a 3 km cycling time trial (Kilding et al. 2012). Interestingly, blinding was weakest post-TTE cycling, potentially due to post-hoc beliefs for treatment efficacy (Table 3). Two participants correctly altered their treatment assignment for NaHCO_3_ capsules from pre-TTE to post-TTE cycling, stating “exercise felt easier/better” as their reason. However, the number of correct ratings for NaHCO_3_ and the cornflour placebo administered in capsules were still less than by chance alone (both treatments 33%), indicating that blinding remained effective. Administering NaHCO_3_ in capsules may improve the scientific rigour of RCTs, allowing researchers to attribute any changes in performance to the treatment instead of other biases, such as placebo effects and/or self-report bias.

Administration approach also impacted the palatability of treatments during study 2, with participants suggesting NaHCO_3_ given in solution was less palatable than the other three treatments (Fig. 1 A). Most participants stated that NaHCO_3_ solution treatments had a ‘bad taste’, although no difference was observed between scores for how likely they would be to use treatments if they knew it would improve their performance (Fig. 1 B). Since athletes believe ergogenic aids provide them with a small competitive edge (Maughan et al. 2007), they might be willing to tolerate poor tasting beverages. Whilst this was the first study to examine differences in palatability between solution and capsule NaHCO_3_ ingestion strategies, it has been reported that the size of capsules (3 vs. 0 vs. 000) does not impact palatability of NaHCO_3_ (Middlebrook et al. 2021). Considerable effort has been made to refine NaHCO_3_ supplementation (i.e., mitigating side-effects), therefore strategies that improve palatability should increase the likelihood of athletes using NaHCO_3_. Our findings suggest that consuming NaHCO_3_ in capsules improves palatability, but practitioners need to consider the trade-off between palatability and practicality due to the high number of capsules (~ 25–30 for a 70 kg athlete) required to achieve a potentially ergogenic dose.

The presence or absence of gastrointestinal discomfort was a common factor referenced by participants when identifying treatments during study 2, as some successfully associated side-effects with a ‘real’ treatment. In agreement with Foad et al. (2005) examining caffeine blinding, participants experiencing side-effects tended to alter treatment assignment towards NaHCO_3_, or vice versa for placebo when they had minimal symptoms (Table 3). Administering NaHCO_3_ in capsules may have improved blinding by reducing severity of gastrointestinal discomfort (Carr et al. 2011), but even mild symptoms could break blinding, in turn causing researchers to overestimate efficacy of NaHCO_3_. Alternatively, participants’ awareness that they have not received a ‘real’ treatment due to an absence of side-effects may mitigate potential ‘expectancy’ effects during deception studies (Hurst et al. 2019; McClung and Collins 2007; Clark et al. 2000). Therefore, it is imperative that researchers adopt ingestion strategies that adequately disguise the placebo, ensuring participants are unable to identify the absence of a ‘real’ treatment.

### Limitations and future research directions

A limitation of this study is that fizziness was not matched when attempting to assess blinding efficacy. Preparing sodium chloride placebo solution treatments with carbonated water (instead of still water) could have allowed us to replicate the fizzy sensation of NaHCO_3_ (Higgins and Shabir 2016) and in turn improve blinding efficacy. Given that consumption of carbonated beverages can increase the likelihood of gastrointestinal side-effects such as satiety and belching (Cuomo et al. 2009), it is possible that preparing sodium chloride placebo treatments with carbonated water would further improve blinding efficacy by inducing similar gastrointestinal distress associated with NaHCO_3_. To note, only 2/12 participants reported ‘fizziness’ upon ingesting the NaHCO_3_ solution in study 2, however, both correctly identified the NaHCO_3_ treatment. It is intuitive to suggest that further work might increase the blinding efficacy of NaHCO_3_ and sodium chloride placebo solution treatments with the use of carbonated water. Future research should also investigate blinding efficacy of NaHCO_3_ administered in smaller doses (0.2 g·kg^−1^ BM) or within different types of capsules (delayed-release, enteric-coated), as these ingestion strategies may reduce severity and occurrence of gastrointestinal side-effects (Gurton et al. 2020; Hilton et al. 2020) which should strengthen blinding during RCTs examining sports performance.

## Conclusion

Given that an overestimation of the efficacy of NaHCO_3_ on sport performance outcomes could occur if researchers do not adequately blind treatments during randomised placebo-controlled trials, it is important that participants are unaware of what they have received. This multi-study project is the first to determine the most efficacious blind for NaHCO_3_ ingestion and provides important implications for researchers aiming to examine its influence on sport performance. Our findings suggests that administering NaHCO_3_ in capsules is the most efficacious blinding strategy during randomised controlled trials, as this resulted in the most unsure ratings for which treatment had been received. In cases where researchers opt to administer NaHCO_3_ via a solution ingestion strategy (i.e., concerns over the high number of capsules required to achieve an ergogenic dose), they should consider flavour and temperature of solutions, with 5 mL·kg^−1^ body mass of chilled (~ 12 °C) orange squash and mineral water identified as optimal ingestion strategy for taste-matching NaHCO_3_ and a sodium chloride placebo. Finally, future research should use carbonated water to improve the blinding efficacy between NaHCO_3_ and placebo in an attempt to further improve the blinding.
